# Automatic Microplot Localization Using UAV Images and a Hierarchical Image-Based Optimization Method

**DOI:** 10.34133/2021/9764514

**Published:** 2021-12-08

**Authors:** Sara Mardanisamani, Tewodros W. Ayalew, Minhajul Arifin Badhon, Nazifa Azam Khan, Gazi Hasnat, Hema Duddu, Steve Shirtliffe, Sally Vail, Ian Stavness, Mark Eramian

**Affiliations:** ^1^Department of Computer Science, University of Saskatchewan, Saskatoon, Saskatchewan, Canada; ^2^Department of Plant Sciences, University of Saskatchewan, Saskatoon, Saskatchewan, Canada; ^3^Agriculture and Agri-Food Canada, Saskatoon, Saskatchewan, Canada

## Abstract

To develop new crop varieties and monitor plant growth, health, and traits, automated analysis of aerial crop images is an attractive alternative to time-consuming manual inspection. To perform per-microplot phenotypic analysis, localizing and detecting individual microplots in an orthomosaic image of a field are major steps. Our algorithm uses an automatic initialization of the known field layout over the orthomosaic images in roughly the right position. Since the orthomosaic images are stitched from a large number of smaller images, there can be distortion causing microplot rows not to be entirely straight and the automatic initialization to not correctly position every microplot. To overcome this, we have developed a three-level hierarchical optimization method. First, the initial bounding box position is optimized using an objective function that maximizes the level of vegetation inside the area. Then, columns of microplots are repositioned, constrained by their expected spacing. Finally, the position of microplots is adjusted individually using an objective function that simultaneously maximizes the area of the microplot overlapping vegetation, minimizes spacing variance between microplots, and maximizes each microplot's alignment relative to other microplots in the same row and column. The orthomosaics used in this study were obtained from multiple dates of canola and wheat breeding trials. The algorithm was able to detect 99.7% of microplots for canola and 99% for wheat. The automatically segmented microplots were compared to ground truth segmentations, resulting in an average DSC of 91.2% and 89.6% across all microplots and orthomosaics in the canola and wheat datasets.

## 1. Introduction

Changing climate, increasing population, and demand for high-quality food will challenge producers to grow more food with greater efficiency. A major pillar of the efforts to address this challenge is the selective breeding of higher-yield crop varieties with greater tolerance to extreme environments. Breeding routinely involves assessment of plants from candidate genetic lines to quantify desirable phenotypes. Current methods for assessing plant phenotypes are time-consuming, relying on laborious manual qualitative assessment, and often only consider a subsampling of the plants grown. For in-field trials, where a field area is subdivided into many *microplots*, within which are grown many plants from a single genetic line, breeders must physically visit each microplot and visually assign scores based on qualitative and quantitative traits of plants and rank the plants at multiple stages of development. A new technique known as image-based phenotyping has potential to improve this subjective process [1, 2].

Image-based phenotyping uses machine learning and image processing techniques to obtain quantitative measurements of the structural and functional properties of plants. For in-field trials, growth, health, and physical traits can be quantified from overhead images acquired from cameras mounted on tractors or unmanned aerial vehicles (UAV). Aerial imaging has potential for faster and more accurate assessment of a field [1, 3]. A camera-equipped UAV typically collects many high-resolution, overlapping aerial images of small sections of the field. Using software, aerial images are stitched together to generate an orthomosaic image, providing a complete view of all the microplots in the field in a single image. Most subsequent analysis relies on knowing the precise locations and identities of each microplot in the orthomosaic image [4, 5]. It is this problem that we study in this paper since localizing microplots in the orthomosaic image is a fundamental first step for any kind of image-based microplot-level phenotype analysis.

Microplots may or may not be regularly shaped or aligned due to inaccuracy during planting/seeding. Even if they are aligned, for example, in a grid-like arrangement (which is common), the orthomosaic may be warped due to small, cumulative errors in the stitching process or inaccuracies in geolocation information, which makes microplot localization more challenging. For example, such warping can make it difficult or impossible to rely on an expectation that microplot boundaries are perfectly straight lines.

Microplots can theoretically be extracted from UAV orthomosaics based on GPS data alone. Usually, this means combining GPS data derived from the orthomosaic with preestablished GPS-based microplot shapefiles. However, both GPS sources are not always present. Or depending on the precision of GPS systems, the two GPS sources could disagree, sometimes in a nonlinear way, by 10 meters or more [6]. Finally, as the growing season progresses, microplots can grow beyond their preestablished shapes in unpredictable ways. Taken together, these factors make it difficult to propagate known plot locations from one orthomosaic image to another of the same field taken later or earlier in the season, necessitating an approach to microplot extraction based on image analysis rather than on GPS alone.

Automated detection and segmentation of microplots would enable automatic monitoring and quantification of microplot phenotypes, allowing a faster selection process that requires much fewer person-hours than manual assessment.

There are three general approaches used to extract microplots from orthomosaic images such as manual, semiautomatic, and automatic methods.

The first method is the manual method that localizes a microplot using the geolocation information on the drone to generate bounding boxes generated geometrically. Haghighattalab et al. implemented a simple grid-based method to extract microplots [7]. As this method was based on the assumption of a fixed microplot boundary size, the polygon boundary may overlap part of the adjacent microplots. This method is easy to implement and fast, but it did not account for gaps between microplots and gaps between each range of microplots.

The semiautomatic approach partially automates plot localization but still requires some manual intervention. Hearst and Cherkauer proposed a framework to extract microplots based on their map coordinates [8]. This approach used Ground Control Points (GCP) and depends on geolocation accuracy of imagery. Their results might also be affected if there is little spacing between microplots or when spaces between crops are obscured by plants in the later stages of growth. Haghighattalab et al. extracted microplots from wheat breeding nursery images [7]. Their semiautomatic algorithm localizes microplots by classifying pixels of images into “vegetation” and “soil” areas. Similarly, Recio et al. proposed a microplot-based image processing method for automated extraction of trees [9]. They used the *K*-means classification algorithm to classify image pixels into “tree” and “nontree” groups. The limitations of both approaches are that they assume ideal separation between plots and sometimes merge multiple microplots into a single microplot. Khan and Miklavic proposed a semiautomated approach to extract microplots from wheat field orthomosaic images [10]. They used particle swarm optimization to find the optimal alignment of a bounding box grid. This method requires a software tool to manually create a cellular grid based on the number of rows and columns laid over the image. Moreover, the method has been tested with only one type of crop and 60 very clearly separated microplots. For many crops and field trial layouts, monitoring the complete growing season requires a robust method to capture images with little to no visible ground between microplots. Tresch et al. developed a semiautomatic approach to obtain microplot information for drone imagery of whole fields using the microplot extraction method known as EasyMPE (microplot extraction) [11]. They were able to identify crop rows and columns in soybean and sugarbeet crops on six different field datasets. However, their method was designed for midseason images in which there is a visible gap between microplots. Their technique might not work for later-season images when plants are larger and such gaps may not exist. Matias et al. developed an R package called FIELDimageR software to analyze many microplots in orthomosaic images [12]. The software was able to crop microplot area, count the number of plants per microplot, and measure canopy cover percentage, vegetation indices, and plant height. Although the software was user-friendly and able to analyze orthomosaic images, the first stage of the software to extract the microplots was semiautomatic. Robb et al. proposed an image processing method to segment crop microplots from aerial imagery [13]. The proposed method was based on identifying the division between microplots to demarcate them. This paper provided a consistently high-performing approach for delineating crop microplots and used minimal input from the user. In general, existing semiautomatic methods have several drawbacks: they require a tool to create a cellular grid based on the number of rows and columns laid over the image, the grid's position and attributes are only obtained using external software, and it is only used with one kind of plant and a limited number of clearly delineated microplots. Furthermore, the semiautomatic methods often assume a rectangular grid and are not easily generalizable to nonrectangular arrangements of microplots.

The third approach is automatic methods, which are fully automatic without any manual intervention required. Parraga et al. proposed a segmentation methodology based on image processing techniques using UAV imagery. The proposed segmentation method had four steps: preprocessing, filtering, ROI map, and validation. This method can automatically segment the wheat microplots during the whole growth stage. However, it needs to tune several variables to work for all images during the whole wheat cycle, which results in a long time run. Ahmed et al. proposed an algorithm to detect and segment lentil microplots from multispectral aerial images [14]. One of the advantages of this approach is that the proposed method is fully automatic. However, this method does not correctly work in late-season images because neighbouring microplots grow into each other. Also, it is unable to distinguish lentil microplots from the guard microplots. Chen and Zhang developed a Python package, GRID (GReenfield Image Decoder), to overcome the limitation of identifying pixel of interest (POI) and microplot segmentation [15]. GRID was able to detect different field layouts such as microplots arranged in rhombus or grid and obtained better performance compared to other software programs with higher computing time. This tool had some limitations. First, the proposed tool is unable to distinguish POI regions if weeds are nearly identical to the vegetation of interest. Second, GRID uses visible channels RGB to control noise and shade and is invalidated for other multispectral images. Despite advances over previous automatic approaches, these methods have some drawbacks. Multiple microplots may be seen as a single microplot, and ideal separation is assumed between microplots. The proposed methods used midseason images that have well-spaced microplots and needed tuning several variables to work for all images.

To the best of our knowledge, all of the published works on microplot extraction have been developed using learning-based and image-based analysis approaches. Although some models are automatic, all models have been designed and tested with orthomosaics having a relatively small number of microplots and used midseason orthomosaic images where there are no canopy closure and relatively uniform appearance of vegetation within microplots and clear gaps in between microplots. Some methods require a tool to manually create a cellular grid [10]. Among the aforementioned approaches, developing an automated method to test with a wider range of crops and growth stages and combine a priori knowledge of the trial layout (initial shapefile) with an optimization approach to adjust the microplot boundaries based on image analysis of the vegetation and soil is needed. Therefore, developing an automatic method where a map of the known microplot layout is overlaid and positioned using learning-based or image-based methods is necessary. An approach to automatically identify microplots from orthomosaic images in a broader range of growth stages is needed. Overlaying a map of the known microplot layout is a good starting point, but the precise boundaries of each microplot need to be refined due to the aforementioned problems of warping and geolocation errors. Clicking four corner points of a rectangular area to initialize an algorithm assumes a rectangular grid layout, even though some trials may have more unequal number of plots in rows or columns due to irregular field shapes. Our main objective is to develop a robust method for the accurate extraction of microplots that can be used in high-throughput image-based plant phenotyping pipelines from aerial drone-acquired images of fields with a greater diversity of plot layouts.

In this paper, we propose a three-step image-based optimization approach where a map of the known microplot layout is overlaid and positioned as optimally as possible. In the next step, the position of individual microplots is optimized within the constraints of the overall layout. Compared to existing methods, ours adds robustness to challenging situations where there are a lack of visible microplot spacing and a lack of no canopy closure. Our approach also only requires a to-scale microplot layout map as input. The three-stage optimization procedure proceeds in a course-to-fine manner from overall field blocks to groups of microplot columns and finally to individual microplots to mitigate the effects of orthomosaic warping.

The rest of the paper is organized as follows. In Section 2, we describe the datasets for wheat and canola, present the microplot localization algorithm, and evaluate algorithm performance.  Section 4 discusses the detection and segmentation results of the proposed algorithm. Finally, Section 5 ends the paper with a short summary and conclusion.

## 2. Material and Methods

### 2.1. Dataset Description and Image Acquisition

**Figure 1:**
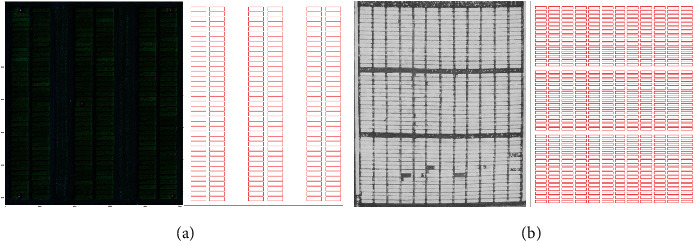
Image samples and scaled microplot maps: (a) an RGB orthomosaic and scaled microplot map for the canola trial in the summer of 2017; (b) an NDVI image and scaled plot micromap for the wheat trial in the spring of 2018.

The datasets used in this paper were obtained from two wheat and canola breeding trials. To capture canola images, a Draganfly X4P quad-copter (Draganfly Innovations Inc., Saskatoon, SK, Canada) carrying a MicaSense RedEdge camera (Micasense Inc., Seattle, WA, USA) was used to take images in the summer of 2017. This camera captures images with five spectral channels: red, blue, green, near-infrared, and red-edge. Images were taken at a height of 15 meters or 20 meters depending on the day for canola datasets. The canola orthomosaic images are about 8100 × 9000 pixels in size. The canola trial has 39 rows and 6 columns of microplots in three aligned blocks of two columns. The microplot dimensions were 5 × 20 ft with an intermicroplot gap of 1 ft between microplots in the same block. A Sentera double 4K NDVI camera was used to take images of wheat at a height of about 19 meters in the spring of 2018. This camera captures NDVI images. The wheat orthomosaic images are about 12000 × 14000 pixels and contain 47 rows and 12 columns of microplots in three aligned blocks of 15, 15, and 17 rows. The wheat microplot dimensions are 3.5 × 13.5 ft with an intermicroplot gap of 2 ft between microplots in the same block. A high-resolution orthomosaic image of each field was produced by stitching images with Agisoft Metashape (Agisoft LLC, St. Petersburg, Russia).  Figure 1 shows the RGB orthomosaic from canola and the NDVI image from wheat breeding trial fields in the summer of 2017 and spring of 2018, respectively.

### 2.2. Ground Truth

Microplot ground truth bounding boxes were manually annotated in the orthomosaic images by a lab technician. A grid of equal-size rectangles corresponding to the known microplot layout was overlaid on the orthomosaic image so that each microplot is near its correct position. A lab technician used LabelImg to create the rectangles around the microplots. The bounding boxes were created by clicking two opposite corner points. Once done, the annotations were saved as xml in pascal voc format.

### 2.3. Microplot Localization Algorithm

#### 2.3.1. Algorithm Inputs

Our method requires the following inputs:

- The orthomosaic image in which microplots are to be localized
- The horizontal and vertical scale of the orthomosaic image in pixels per meter
- A to-scale *microplot map* of the microplot layout in the field in the form of a list of lists of coordinates of corner points of the microplot bounding boxes in units of meters relative to an arbitrary origin

The scale of the orthomosaic image is determined by the commercial software used to stitch the orthomosaics. The microplot map is easy to produce from the known field layout. It is usually convenient to use the top-left-most microplot corner as (0, 0). The list of lists of microplot coordinates is ordered so that microplots can be easily associated with their microplot IDs in the field trial design. In the case of our regular-grid field layouts, the microplots are ordered in a column-major fashion.

#### 2.3.2. Preprocessing and Map Scaling

RGB orthomosaic image inputs are converted to a grayscale vegetation index image using an index that produces larger values for vegetation than soil. There are a few vegetation indices that satisfy this requirement such as NDVI, Excess Green (ExG), or Excess Green minus Excess Red (ExG-ExR) used by [16, 17]. Indeed, any vegetation index that behaves similarly could be used. We used ExG for our canola orthomosaic images. Grayscale orthomosaic image inputs are assumed to already be a suitable vegetation index image. In the case of our wheat images, the camera used captured NDVI images directly, which we used as input. It is assumed that the orthomosaic is oriented such that the long edges of microplots are parallel to the horizontal image coordinate axes.

The input scales of the orthomosaic image are used to scale the microplot map so that it is the correct size within the orthomosaic image's coordinate system by multiplying map's horizontal and vertical coordinates by the image's horizontal and vertical scale in pixels per meter, respectively.

#### 2.3.3. Per-Block Optimization

The next step of the algorithm is to find a translation of the scaled microplot map that maximizes the amount of vegetation within the microplot areas. Since the convex hulls of our microplot maps are rectangular, we searched for a translation that maximizes the sum of the vegetation index within the entire microplot map's bounding rectangle. For more irregularly shaped microplot maps or orthomosaics where stitching-induced warping would make successful alignment of the entire microplot map difficult, one could divide the microplot map into a small number of *blocks* and find a translation for the initial alignment of each block independently. In general, let *B* be a set of minimum bounding boxes around blocks of microplots, *b* + *t* be the translation of some *b* ∈ *B* by *t* = (Δ*x*, Δ*y*), and veg(*b*) be the sum of the vegetation index image within a rectangle *b*. The objective function to be maximized is

(1) $$\begin{array}{c} \phi_{\text{veg}}\left(B,t\right)=\frac{\sum_{b\in B}\text{ veg}\left(b+t\right)}{\text{area}\left(B\right)}, \end{array}$$

where *b* + *t* is the rectangle *b* translated by *t* and area(*B*) is the number of pixels enclosed by all rectangles in *B*. As long as the vegetation index ranges between 0.0 and 1.0, *ϕ*_veg_(*B*, *t*) will has a value in the same range.

**Figure 2:**
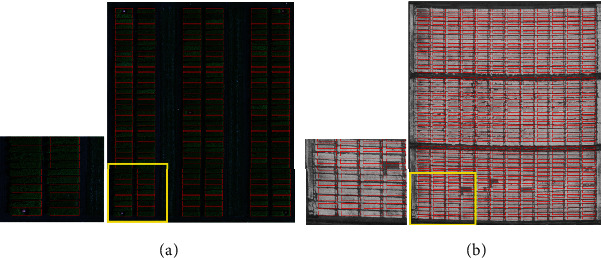
Example of microplot map overlay after a per-block optimization method. The zoomed-up areas show that not all plot boxes may be well-aligned due to the orthomosaic warping.

The *t* that optimizes this objective function is found using differential evolution (DE) [18]. It is an evolutionary algorithm, which is able to find the minimum of a function *f*(*x*): *IR*^*n*^⟶*IR* without requiring its derivative. Since this objective function and objective functions in subsequent algorithm steps are composed of nondifferentiable functions, DE was an ideal optimization algorithm for our task.  Figure 2 shows the “optimal” translation of the microplot maps.

#### 2.3.4. Per-Column Optimization

As can be seen in the magnified areas of the orthomosaics in Figure 2, the initial placement of the blocks does not always align every microplot rectangle perfectly with microplots in the orthomosaic. Such misalignment is caused by warping of the orthomosaic which occurs during the stitching process. Some misalignment caused by warping can be reduced by dividing the microplots into more than one block and performing the previously described initialization step independently on each block. In any case, the second phase of our proposed algorithm repositions each column independently within constraints of the expected microplot map to mitigate these effects. This is done across all blocks rather than for each block independently to take advantage of the overall grid-like layout of microplots.

For each column, we let *B* be the bounding box for the entire column and optimize equation (1) using DE to find a translation vector *t* so that *B* encloses a greater sum of excess green. Then, each individual rectangle in the column is translated by *t*. This works for most orthomosaics, but we observed that this can reposition columns such that they are overlapping. This occurs in some late-season images where there is canopy closure between neighbouring columns with no visible ground. This is corrected in a second pass through the columns where for each column *c* (except for the right-most column), if the bounding box of *c* overlaps that of the column to its right, *c*′, then *c* is translated to the left so that it is positioned *s* pixels to the left of *c*′ where *s* is the number of pixels between *c* and *c*′ in the initial scaled microplot map. The per-column optimization process is described in Algorithm 1.

For microplot maps which are not organized into equal length columns, this step could be generalized and be applied to any set of subsets of microplots *B*_1_, *B*_2_, ⋯, *B*_*n*_ and their bounding boxes or convex hulls with varying degrees of ease depending on the complexity of the spatial relationships between the subsets of microplots. For subsets with rectangular bounding boxes in a grid-like arrangement, the generalization is fairly easy. Moreover, this step could equivalently be performed as a per-row optimization.

#### 2.3.5. Per-Microplot Optimization

While the per-column optimization step somewhat mitigates the orthomosaic warping problem, individual microplots in a column can still be positioned poorly especially if the warping causes vertical displacement.

The per-microplot optimization method requires three steps. The complete steps are summarized in Algorithm 2.

*(1) Update Initial Points*. Individual microplot positions are optimized in a column-major order, starting from the top-left microplot, then working down the column, and starting at the top of the next column to the right when the current column has been completed. The differential evolution (DE) method is a population-based stochastic method that needs two important parameters for optimization, initial points and bounding research. In our project, the initial points are the upper-left corner and the lower-right corner of each bounding box. To update the initial points, the location of the optimized microplots from the previous column and the location of the previous microplot in the same column are needed. Let (*r*′_1_, *c*′_1_) and (*r*′_2_, *c*′_2_) be the upper-left corner and the lower-right corner of all microplot rectangles, respectively.

(2) $$\begin{array}{c} g_{d}=\text{microplo}{\text{t}}_{A.2}\left(r_{2}^{\prime }\right)-\text{microplo}{\text{t}}_{A.1}\left(r_{2}^{\prime }\right). \end{array}$$

**Figure 3:**
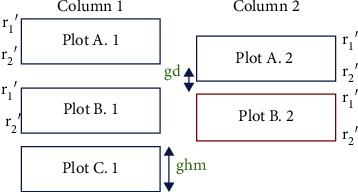
The blue bounding boxes show the optimized microplots. The red bounding box shows the current microplot that we want to update its initial points.

Let *g*_*hm*_ be the height of microplot and the *g*_*d*_ shows the difference between two microplots in the same row but in a different column that can show the amount of warping from the previous column. By observing the warping between two neighbouring microplots in the same row, the amount of displacement due to warping is equal to or less than *g*_*hm*_/2. Then, initial points are updated when *g*_*d*_ < = (*g*_*hm*_)/2. As can be shown in Figure 3, updated initial points for microplot_*B*.2_ are ((microplot_*B*.1_(*r*′_1_) + *g*_*d*_), *c*′_1_) and ((microplot_*B*.1_(*r*′_2_) + *g*_*d*_), *c*′_2_). If *g*_*d*_ > (*g*_*hm*_)/2, it is more likely that the optimized microplot from the previous column was aligned more closely or exactly to its neighbouring microplot. Therefore, the initial points are not updated. In addition, the initial points of microplots in the first column and the first row of microplots in the microplot map are not updated because there is no information from their previous microplots.

*(2) Optimize the Location of Microplot*. In this stage of our algorithm, the position of individual microplots in the microplot map is adjusted by optimizing equation (1) to achieve the best possible overlap with the corresponding microplot in the orthomosaic. For this phase, we formulated an objective function consisting of five components using the underlying microplot layout and image characteristics while retaining the general principle of maximizing overlap with plant areas:

(3) $$\begin{array}{c} \phi \left(t\right)=w_{0}\cdot \left(1-\phi_{\text{veg}}\left(t,B\right)\right)+w_{1}\cdot \phi_{\text{EC}}\left(g_{k}\right)+w_{2}\cdot \phi_{\text{AC}}\left(r_{1},r_{2}\right)+w_{3}\cdot \phi_{\text{TC}}\left(t\right)+\phi_{\text{EdC}}\left(c_{\min},r_{\min}\right), \end{array}$$

where *t* = (Δ*x*, Δ*y*) is a translation vector, *B* is the bounding box of the microplot whose position is to be optimized, *w*_0_, *w*_1_, ⋯, *w*_5_ are weighting terms (*w*_4_ and *w*_5_ are embedded in *ϕ*_EdC_, equation (9)), and the remaining parameters to the individual components are functions of *t* and are described in the following paragraphs.

Individual microplot positions are optimized in a column-major order, starting from the top-left microplot, then working down the column, and starting at the top of the next column to the right when the current column has been completed. The microplot position is optimized using DE to find the *t* that minimizes this function. The search space of the optimization is bounded such that Δ_*x*_ is between Δ*x*_min_ and Δ*x*_max_, and Δ*y* is between Δ*y*_min_ and Δy_max_. In our implementations, we use bounds dependent on *g*, the known gap size in pixels between adjacent microplots in the initial scaled mircoplot map.

The first term in the cost function is 1 − *ϕ*_veg_(*t*, *B*) (equation (1)) which is minimized when the sum of the vegetation index within the microplot is maximized (for our datasets, the index is ExG for canola and NDVI for wheat).

The next term is the even spacing cost (EC). It is minimized when the vertical gap between a microplot and its previously optimized neighbour equals the expected gap *g* based on the initial field map. For microplots at the top of the column that have no previously processed neighbour above them, the EC term is not used. Let *g*_*k*_ = *g*_*i*_ + Δ*y* be the current gap between the current microplot *p* and the previously optimized microplot *p*′ above it, where *g*_*i*_ is the initial gap after the optimization of *p*′'s position, but before the commencement of the optimization of *p*'s position. Let $\overset{¯}{g}$ be the normalized vertical gap between the microplot being optimized and microplot above it in the column:

(4) $$\begin{array}{c} \overset{¯}{g}=\frac{\;|\;g_{k}-g\;|\;}{\max\left(\left|\Delta y_{\min}\right|,\left|\Delta y_{\max}\right|\right)}. \end{array}$$

The even spacing cost (EC) is then defined as

(5) $$\begin{array}{c} \phi_{\text{EC}}\left(g_{k}\right)=1-\exp\left(-4\overset{¯}{g}\right). \end{array}$$

The search space for Δ*y* is bounded by the expected vertical gap between microplots *g* so that the optimizer can move the bounding box at most *g* pixels up or down. Therefore, we normalized ∣*g*_*k*_ − *g*∣ based on the difference between the maximum possible gaps between the positions of two estimated bounding boxes. We apply an exponential function to this to get *ϕ*_EC_(*g*_*k*_). An exponential cost was selected instead of a linear cost to obtain a higher cost value for small normalized gaps (errors) in spacing.

The third term is the alignment cost (AC). It is minimized when the centers of the microplots in a column are horizontally aligned. Let the starting center of the current microplot rectangle be (*r*_1_, *c*_1_) and (*r*_2_, *c*_2_) be the center of the previously processed microplot rectangle above it. Then, the alignment cost (AC) is

(6) $$\begin{array}{c} \phi_{\text{AC}}\left(r_{1},r_{2}\right)=\frac{\left|r_{1}+\Delta x-r_{2}\right|}{\max\left(\left|\Delta x_{\min}\right|,\left|\Delta x_{\max}\right|\right)+\left|r_{1}-r_{2}\right|}. \end{array}$$

A linear cost here yielded a better result than a nonlinear cost because gaps between columns, especially in the canola images, are larger than the gap between microplots within a column. Thus, the penalty for smaller deviations needed to be lessened in comparison to the evenness cost.

The fourth term is the translation cost (TC) which encourages translation vectors with shorter length. It is minimized when *t* = (Δ_*x*_, Δ_*y*_) = (0, 0). This is desirable since we can be reasonably certain at this phase of the algorithm that microplots in the microplot map are already quite close to their correct position. Translation cost is defined as the sum of the normalized Manhattan distance of the shift.

(7) $$\begin{array}{c} \phi_{\text{TC}}\left(t\right)=\frac{\left|\Delta_{x}\right|+\left|\Delta_{y}\right|}{\max\left(\left|\Delta x_{\min}\right|,\left|\Delta x_{\max}\right|\right)+\max\left(\left|\Delta y_{\min}\right|,\left|\Delta y_{\max}\right|\right)}. \end{array}$$

**Figure 4:**
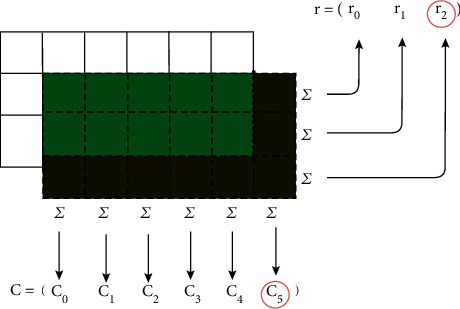
Pixels of a microplot are illustrated with solid lines whereas the estimated bounding box is depicted using dashed lines. Green and brown colors are used to demonstrate where the bounding box overlaps with the microplot (vegetation) or soil. *c*_*i*_ and *r*_*i*_ are the row and column sums of *M*, with *c*_5_ and *r*_2_ being the minimum row and column sums, respectively.

The last term in the objective function is the edge cost (EdC). This term encourages microplots to be nudged away from positions where the very edge of the microplot area overlaps nonmicroplot areas in the orthomosaic. It is illustrated in Figure 4. Let *M* be the subimage of the vegetation index orthomosaic enclosed by the microplot's bounding box with width *w* pixels and height *h* pixels. The edge cost is minimized when the sum of the smallest row sum and smallest column sum of *M* is maximized. Let *M*_*ij*_ be the vegetation index of the pixel at the *i*-th row and *j*-th column of the subimage *M*. The smallest row and column sums are

(8) $$\begin{array}{c} c_{\min}=\underset{j}{\min}\frac{1}{h}\overset{i=h}{\underset{i=1}{\sum }}M_{ij},\\ r_{\min}=\underset{i}{\min}\frac{1}{w}\overset{j=w}{\underset{j=1}{\sum }}M_{ij}, \end{array}$$

where 1/*h* and 1/*w* are normalizing factors. In the example in Figure 4, the bottom-most row and right-most column have the smallest vegetation index because the microplot is misaligned so the bottom row and right column overlap the ground rather than vegetation. Thus, *c*_5_ and *r*_2_ are the minimum column and row sums, respectively. The edge cost to be minimized is

(9) $$\begin{array}{c} \phi_{\text{EdC}}\left(c_{\min},r_{\min}\right)=w_{4}\cdot \left(1-c_{\min}\right)+w_{5}\cdot \left(1-r_{\min}\right). \end{array}$$

where *w*_4_ and *w*_5_ are the remaining weighting terms omitted from equation (3).

**Figure 5:**
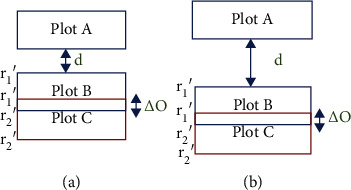
The blue bounding boxes show the optimized microplots. The red bounding box shows the currently optimized microplot. (a) The current microplot is overlapped with the previously optimized microplot. (b) The previously optimized microplot is overlapped with the currently optimized microplot.

*(3) Find Overlapped Microplot and Omit It -1.2 cm*. In this stage of per-microplot optimization algorithm, the overlapped microplot in the field is found by using information from the previous optimized microplot. The reason for adding this stage to the algorithm is that initial points for the first column and first row of individual microplots in the microplot map are not updated. Since the initial points and research areas play a vital role in optimizing the location of microplots, there is a high possibility of overlapping between microplots in the first column. Two kinds of overlapping may occur after optimizing the location of microplot. First, the current optimized microplot's location is overlapped with the previous microplot; second, the previously optimized microplot is overlapped with the currently optimized microplot.  Figure 5 shows two examples of overlapped microplots in the field.

(10) $$\begin{array}{c} \Delta O=\text{microplo}{\text{t}}_{B}\left(r_{2}^{\prime }\right)-\text{microplo}{\text{t}}_{C}\left(r_{1}^{\prime }\right). \end{array}$$

Δ*O* is defined as the height of the overlapped area between two microplots. Microplot *A* and microplot *B* are two previously optimized neighbours. If the distance *d* is equal or less than expected gap *g* based on the initial map, it means that the current microplot overlapped with its previous neighbour. Then, to omit the overlapping problem, microplot_*C*_(*r*′_1_) + Δ*O* and microplot_*C*_(*r*′_2_) + Δ*O*. When the distance *d* is more than the expected gap *g*, it means that the previously optimized microplot overlapped with the current microplot. Then, to omit the overlapping problem, microplot_*C*_(*r*′_1_) − Δ*O* and microplot_*C*_(*r*′_2_) − Δ*O*.

The complete algorithm is summarized in Algorithm 3.

### 2.4. Hyperparameter Tuning

We performed a hyperparameter tuning step that optimizes *w*_0_, *w*_1_, ⋯, *w*_5_ to accommodate seasonal variations in the microplot orthomosaic. We used DE for the hyperparameter search. For each dataset, we randomly selected one early-, one mid-, and one late-season image on which to perform the hyperparameter search. The objective function for this DE optimization was the mean DSC of the three images after segmentation with our algorithm, and *w*_0_, *w*_1_, ⋯, *w*_5_ are the variables being optimized. For the DE algorithm, the minimum and maximum bounds on the variables were set to 0.0 and 1.0.

The values of the hyperparameters used for evaluation are reported in Section 2.5.3.

### 2.5. Algorithm Performance Evaluation

We assessed the algorithm's ability to detect microplots and its ability to segment microplots accurately.

#### 2.5.1. Detection Metrics

A microplot is considered *detected* if the overlap between the microplot and its ground truth is acceptably high. We quantified this overlap using the Dice Similarity Coefficient (DSC) [19]. If *A* is the set of pixels in a ground truth microplot and *B* is the set of pixels in the corresponding automatically segmented microplot, then the DSC is

(11) $$\begin{array}{c} \text{DSC}=\frac{2\left|A\cap B\right|}{\left|A\right|+\left|B\right|}. \end{array}$$

Thus, a microplot can be considered *detected* if its DSC exceeds some threshold *T*_DSC_. DSC ranges between 0.0 (no overlap at all) and 1.0 (perfect overlap).

#### 2.5.2. Segmentation Metrics

To evaluate the accuracy of the segmentation, we determined the DSC for each microplot (including microplots not deemed to be detected). We also determined the *displacement error* for each microplot, defined as the Euclidean distance between the center point of the segmented rectangle and its corresponding ground truth bounding box. This metric is used by [10].

In order to facilitate comparison with future studies, we also quantified the segmentation accuracy in terms of sensitivity-specificity and precision-recall. Sensitivity, specificity, precision, and recall are quantities that are computed from the following counts:

- True Positive Pixels (TP): number of pixels correctly labeled as belonging to the correct microplot
- True Negative Pixels (TN): number of pixels incorrectly labeled as belonging to the correct microplot
- False Positive Pixels (FP): number of pixels correctly labeled as background (not belonging to the correct microplot)
- False Negative Pixels (FN): number of pixels incorrectly labeled as background

Sensitivity and specificity are complementary measures. Sensitivity is the proportion of pixels that are part of a microplot that are correctly labeled as such:

(12) $$\begin{array}{c} \text{Sensitivity}\left(\text{Recall}\right)=\frac{\text{TP}}{\text{TP}+\text{FN}}. \end{array}$$

Specificity is the proportion of pixels that are not part of a microplot that are correctly labeled as such:

(13) $$\begin{array}{c} \text{Specificity}=\frac{\text{TN}}{\text{TN}+\text{FP}}. \end{array}$$

The segmentation accuracy is higher when both sensitivity and specificity are close to 1.0. When sensitivity is higher, it means that more of the foreground pixels are being included in the segmentation. When specificity is higher, it means fewer background pixels are being included in the foreground. Both conditions are required for excellent segmentation.

Precision and recall are similar complementary measures in that better segmentations are characterized by both measures being closer to 1.0. Recall is defined in the same way as sensitivity (see above). Precision is the proportion of pixels labeled as microplot that actually do belong to microplots:

(14) $$\begin{array}{c} \text{Precision}=\frac{\text{TP}}{\text{TP}+\text{FP}}. \end{array}$$

The main difference between sensitivity-specificity and precision-recall is that the latter does not take into account TN pixels in the segmentation. Precision-recall therefore does not account for correctly labeled nonmicroplot pixels. However, precision-recall is a good alternative to sensitivity-specificity when the number of background (nonmicroplot) pixels is large relative to the number of foreground (microplot) pixels in an image, which can inflate the specificity score because TN pixels vastly outnumber FP pixels. Thus, we report both pairs of measures.

#### 2.5.3. Evaluation Procedure

Our algorithm was evaluated on the ten canola and seven wheat orthomosaic images described in Section 2.1. The algorithm was implemented in Python on a MacBook Pro (15-inch, 2017) equipped with 2.8 GHz Quad-Core Intel Core i7 CPU (16 G memory).

**Figure 6:**
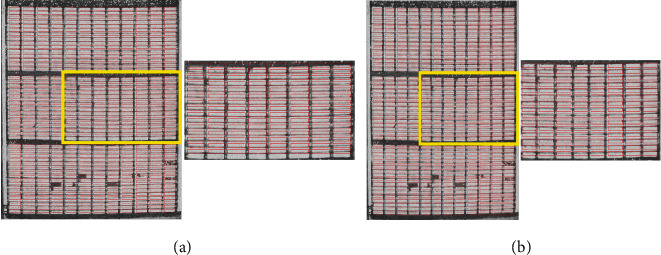
Example result of per-block optimization on the wheat dataset. (a) Result for a single block. The alignment with the middle block is poorly aligned due to orthomosaic warping. (b) Result for three blocks. Greater overlap between the individual position of microplots and the scaled microplot map will result in better microplot segmentation in a per-microplot optimization step.

For the canola dataset, the per-block optimization step was performed using a single block for the entire field. For wheat, the warping was severe enough to require dividing the microplots into three blocks for this step. The blocks were formed from the three obvious subregions in the field with the larger spaces between them as seen in Figure 1(b). Results of the per-block optimization step on the wheat dataset in a single block and three blocks are shown in Figures 6(a) and 6(b), respectively. To assess the benefits of using multiple blocks, we also computed results for the algorithm where all microplots in the wheat orthomosaics are processed as a single block.

The search bounds on *t* for the DE optimizer in the per-block optimization step were set to Δ*x*_min_ = Δ*y*_min_ = 0, Δ*x*_max_ = *g*_*w*_, and Δ*y*_max_ = *g*_*h*_, where *g*_*h*_ is the height of orthomosaic image minus the height of all microplots and the expected distance between them and *g*_*w*_ is the width of the orthomosaic image minus the width of all microplots and the expected distance between columns taken from the initial microplot map for both canola and wheat orthomosaics.

The search bounds on *t* for the DE optimizer in the per-column optimization step were set to Δ*x*_min_ = −*g*_*i*_, Δ*y*_min_ = −*g* and Δ*x*_max_ = *g*_*i*_, Δ*y*_max_ = *g*, where *g* is the expected distance between immediately adjacent microplots and *g*_*i*_ is the expected distance between immediately adjacent columns taken from the initial microplot map for both canola and wheat orthomosaics.

The search bounds on *t* for the DE optimizer in the per-microplot optimization step were set to Δ*x*_min_ = Δ*y*_min_ = −*g* and Δ*x*_max_ = Δ*y*_max_ = *g* for canola orthomosaics. Due to severe warping in the wheat dataset, two search bounds were set. Δ*x*_min_ = Δ*y*_min_ = −*g* and Δ*x*_max_ = Δ*y*_max_ = *g* for microplots in the first row and the first column of microplots in the microplot map in the orthomosaics and Δ*x*_min_ = −*g*, Δ*y*_min_ = −*g*/2 and Δ*x*_max_ = *g*, Δ*y*_max_ = *g*/2 for the rest of microplots in the microplot map in the orthomosaics where *g* is the expected distance between immediately adjacent microplots taken from the initial microplot map.

Hyperparameters used for both wheat and canola datasets were *w*_0_ = 0.976, *w*_1_ = 0.873, *w*_2_ = 0.975, *w*_3_ = 0.0918, *w*_4_ = 0.421, *w*_5_ = 0.822 and *w*_0_ = 0.639, *w*_1_ = 0.627, *w*_2_ = 0.520, *w*_3_ = 0.0118, *w*_4_ = 0.0138, *w*_5_ = 0.624, respectively (rounded to three significant digits).

Because the differential evolution (DE) method is a population-based stochastic method that uses random initializations, we ran our algorithm 5 times for each orthomosaic image and retained the result with the smallest sum of *ϕ*(*t*) over all microplots.

## 3. Results and Discussion

### 3.1. Detection Results

We considered a microplot to be detected with an acceptable overlap if its DSC is 0.5 or greater. It must be understood that this DSC threshold is not used to measure whether a plot is segmented acceptably well, but rather as a measure of the detection rate, that is, the proportion of microplots for which we are able to establish a correspondence with one of the bounding boxes and associate it with a microplot ID. The position of such rectangles is adjusted in a later phase of the algorithm before assessing the quality of the segmentation of the microplot. A large proportion of microplots was detected using this criterion in both the wheat and canola datasets for early-, mid-, and late-season images.  Table 1(b) presents the number of unidentified microplots for each canola orthomosaic image. On average, 99.74% of microplots in an orthomosaic images were detected, an average of 0.6 undetected microplots per orthomosaic.

Table 2(b) reports the same data for the wheat orthomosaics using one block and three blocks for the per-block optimization step. Because of the aforementioned warping, the algorithm detected only 88.55% of microplots in an orthomosaic on average or an average of 64.57 unidentified microplots per image (using the DSC ≥ 0.5 criterion). However, the use of three blocks, which processes the initial microplot map in smaller sections, achieves much improved performance. Table 2(b) shows the results of using three blocks where the average percentage of identified microplots in an orthomosaic improves to 98.99% or an average of 5.7 unidentified microplots per orthomosaic.

### 3.2. Segmentation Results

Tables 1(a) and 2(a) show the mean microplot DSC and median of displacement error after per-block, per-column, and per-microplot optimization steps for our ten canola and seven wheat orthomosaic images. The algorithm achieved an average DSC of 86.58%, 88.34%, and 91.15% across all microplots and orthomosaics tested for the canola dataset after per-block, per-column, and per-microplot optimization steps, respectively. Furthermore, the algorithm achieved an average DSC of 59.04%, 67.67%, and 89.56% across all microplots and orthomosaics tested for the wheat dataset after per-block, per-column, and per-microplot optimization steps, respectively. We present the displacement errors for each of the ten different canola trials in Table 1(a), and the median errors are 0.86, 0.72, and 0.51 ft across all microplots and orthomosaics tested for the canola dataset after per-block, per-column, and per-microplot optimization steps, respectively. In addition, the median errors are 3.76, 2.72, and 0.42 ft across all microplots and orthomosaics tested for the wheat dataset after per-block, per-column, and per-microplot optimization steps, respectively. The mean microplot DSC is significantly increased when three optimization steps are performed for the wheat and canola datasets. In addition, the median of displacement error is reduced compared to performing only one or two optimization steps. The results show the potential advantages of performing a three-level hierarchical optimization method in the presence of low to high severe orthomosaic warping.

Table 1(b) shows the mean microplot DSC (including undetected microplots) for our ten canola orthomosaic images. The algorithm achieved an average DSC of 91.15% across all microplots and orthomosaics tested for the canola dataset. The August 16 image is a late-season image where the gaps between microplots were obscured by vegetation. Although the median of displacement errors and mean of DSC are higher than those of other images, the number of unidentified microplots remained low (Table 1(a)). It is the only canola image where the number of unidentified microplots was greater than 1.

Table 2(b) shows mean DSC and the displacement errors for the seven different wheat orthomosaics when a single block and three blocks are used. The algorithm achieved an average DSC of 88.55% and 98.99% across all microplots and orthomosaics tested for the wheat dataset using single block and three blocks, respectively. The median of displacement error when a single block used is 0.52 ft overall. The warping results in microplot map rectangles being closer to a neighbouring microplot in the orthomosaic than the correct microplot in the orthomosaic. Then, the per-microplot optimization step aligns them more closely to the neighbouring microplot. The spacing between microplots is 2 ft and the width of each microplot is 3.5 ft; thus, a microplot aligning to its neighbouring microplot would cause a displacement error of around 5.5 ft. Table 2(b) shows the median of displacement error when three blocks used are 0.42 ft overall. The number of the undetected microplot is reduced compared to a single block, showing the potential advantages of processing the microplot map in blocks in the presence of orthomosaic warping.

**Figure 7:**
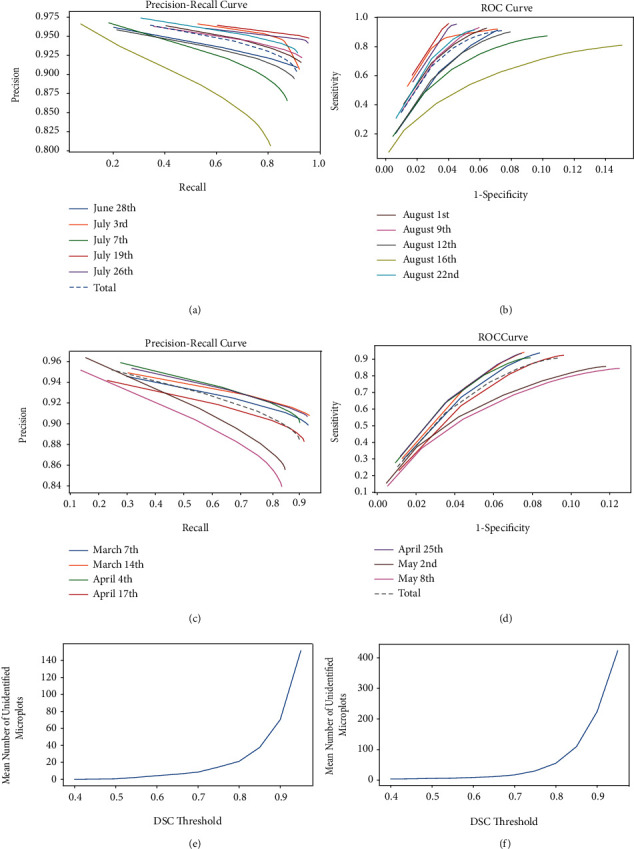
Plot of precision-recall and ROC curves for the canola and wheat datasets. (a) Precision-recall curve for the canola dataset. (b) ROC curve for the canola dataset. (c) Precision-recall curve for the wheat dataset. (d) ROC curve for the wheat dataset. Plot of mean number of unidentified microplots for both (e) canola and (f) wheat datasets.

Figure 7 shows a plot of precision-recall and ROC curves for both canola and wheat datasets, respectively. In Figure 7(a), at recall 0.8, most images have a precision of 0.9 or higher except one image. The August 16 image is a late-season image that the gaps between microplots were covered by vegetation, and the estimated bounding boxes were more likely to be out of position as a result. Figure 7(b) shows that at a specificity of 0.93, the sensitivity of the segmentation is at least 0.8. Again the exception is the August 16 image.

In Figure 7(c), for the wheat images (using 3 blocks), at recall 0.8, most images have a precision of 0.88 or higher except one image. The May 8 image is a late-season image that the gaps between microplots were covered by vegetation. Although the image had a lower precision at recall 0.8, the percentage of the identified microplots for the image was 96.63% from Table 2(b) and 545 microplots were detected (DSC ≥ 0.5) out of 564 microplots. Also, in Figure 7(d), we see that at specificity 0.8, all but one image has a sensitivity of 0.72 or higher. Again the exception is the May 8 late-season images.

Figures 7(e) and 7(f) illustrate how selection of the DSC threshold for microplot detection affects the number of unidentified microplots. In Figure 7(e), at DSC = 0.5, the number of unidentified microplots for the canola dataset is almost zero and does not begin to increase rapidly until the DSC threshold is 0.7 or more. Similar behaviour is observed for the wheat dataset.

**Figure 8:**
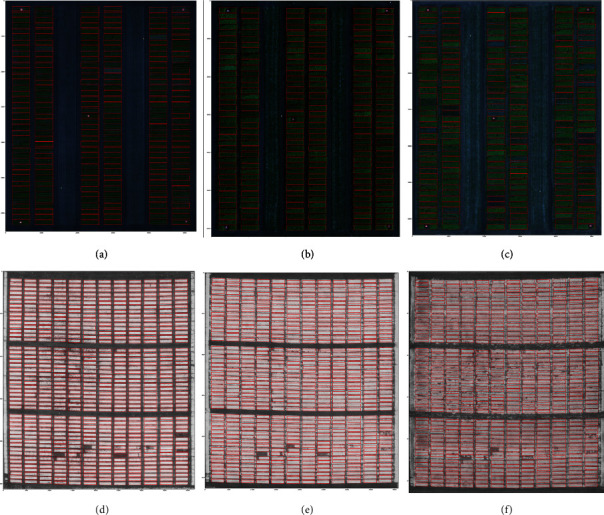
The results of our proposed algorithm on the canola and the wheat datasets. The results on the canola dataset (a) in early-season, (b) midseason, (c) late-season images. The orthomosaic image channels produced by Agisoft PhotoScan contained absolute reflectance values for each of the color band of the RedEdge camera. These reflectance values were not optimized for viewing, so they were brightened by multiplying RGB channel values by a constant factor for reproduction here. The results on the wheat dataset (d) in early-season, (e) midseason, and (f) late-season images.

Figure 8 shows sample segmentations for both crops used in this study including examples of early-, mid-, and late-season orthomosaics. Red rectangles illustrate the final positions of the microplot map rectangles after completion of the algorithm.

## 4. Discussion

We have developed a fully automatic method to extract microplots in orthomosaic images. Our proposed method can be applied to rectangular-shaped microplot layouts. These layouts include rows and columns arranged perpendicularly. Our method works for NDVI and ExG images and allows users to derive the different vegetation indices as input. The most important advantage of this method can be generalized to other crop types as long as the field layout map is provided and plants have a consistent size within a trial. The main difference of our approach with previous methods is combining prior knowledge (shapefile) with image analysis within a multiobjective optimization and evaluating the approach with a wide range of crop type and growth stage. We obtained a better result for the canola dataset compared to the wheat dataset. As shown in Figure 1, there is more misalignment in the wheat dataset caused by the warping of the orthomosaic, which occurs during the stitching process. Furthermore, the wheat dataset has some missing microplots that caused an error while extracting the exact location of microplots.

As shown in Tables 1(a) and 2(a), the mean microplot DSC is significantly increased, and the median of displacement error is reduced after performing each optimization step. It means that each step of optimization is necessary for the presence of low to high severe orthomosaic warping for the wheat and canola datasets.

In this project, initial points and research bounds are essential parameters for the optimization to get the best results. Due to severe warping in the microplot map, some initial points are closer to a neighbouring microplot in the orthomosaic than to the correct microplot in the orthomosaic. Then, the per-microplot optimization step aligns them more closely to the neighbouring microplot. Updating initial points could help to solve an overlap problem and decrease the number of unidentified microplots. To update initial points, the algorithm requires the information of previously optimized microplots. Because of this, the initial points in the first row and column of microplots in the microplot map in the orthomosaic could not be updated. Then, the stage of finding an overlapped microplot was added to the per-microplot optimization step.

The method for extracting and localizing microplots in the field described in this study would benefit any applications that use a remotely sensed field trial, whether for lodging prediction, herbicide tolerance estimation, detecting crop rows, detecting plants, estimating height, or estimating yield [20–22]. For any of these tasks, we must begin by establishing the precise location and area and perimeter of the field's microplots. Although image-based phenotyping performs better than previous manual techniques, there is still substantial labour involved in identifying and segmenting microplots using manual, semiautomatic, and automatic methods. The automatic registration of microplot locations provided by the optimization algorithm proposed here can simplify the tedious preanalysis task of identifying field microplots in an orthomosaic image of a plant research field trial. Our research, by obtaining precise location from microplots, provides a useful and scalable method for high-throughput plant phenotyping scenarios. The outcome of this research will help to increase the throughput of aerial image-based phenotyping.

One limitation of per-microplot optimization used for microplot identification is that our algorithm used NDVI images or ExG indices that provided higher intensity value of vegetation than that of soil. A limitation of finding hyperparameters is that hyperparameter tuning on the whole dataset is quite time-consuming. As such, the hyperparameter tuning method used in this study has used only three images from the dataset to obtain an efficient weight vector. Using a technique such as *k*-fold cross validation on the whole dataset may result in hyperparameter values that better generalize unseen datasets. This could take the form of random splits of a dataset into “training” (for optimizing hyperparameters) and “testing” (for performance evaluation) sets.

In future work, we aim to develop an algorithm that accounts for the different physical properties of various crop types and different microplot sizes. This will be achieved by finding general optimization techniques and making modifications to the objective function that are well suited to manipulate images and vegetation properties. In addition, since the search bounds are important when optimizing the position of blocks, columns, and individual microplots, we suggest including the search bounds in the hyperparameter tuning model. Furthermore, since height information might be helpful for late-season imagery, we could add the height information as another channel to our algorithm. Also, EasyMPE is a semiautomatic approach to obtain microplot information for drone imagery of whole fields [11]. The main part of EasyMPE is the microplot extraction part that is related to our work. This method was designed for midseason images in which there is a visible gap between microplots. In this method, the field is partitioned based on the sum of white pixels in the columns in a segmented binary image. Given the knowledge that we have on late-season and early-season images, it is more likely that this method will not work on these kinds of images. In future work, we could compare our algorithm with the EasyMPE method and apply EasyMPE in late-season and early-season images to ensure our claim.

In some cases, plant senescence or disease may cause microplots to no longer be green, in which case it will be difficult for our algorithm to detect it. Indeed, some examples of this can be seen in the lower block of microplots in Figure 8. We might be able to detect and repair such errors by comparing overlapping microplot rectangles to all four of their expected neighbours in the grid layout. This additional context could be either added as a postprocessing step or incorporated into the objective function of the per-microplot optimization step.

## 5. Conclusion

We have developed an algorithm based on a novel application of image-based optimization techniques to extract and segment microplots within a field. Our proposed method for segmenting wheat and canola trials performs automatic initialization of the known field layout over the orthomosaic images in roughly the right position. The algorithm not only relieves the processing time bottleneck of identifying and segmenting microplots in high-throughput image-based phenotyping pipeline but also can simplify the tedious preanalysis task of identifying microplots in an orthomosaic image. Being able to segment and extract the exact location of microplots for high-throughput plant phenotyping scenarios, such pipelines will be critical in the future search for the higher quality of harvested crop needed to feed a growing population. As the three-stage optimization procedure proceeds in a course-to-fine manner from overall field blocks, it has the potential to be adapted for more specific purposes, such as extracting the different physical properties of various crop types and different microplot sizes.

## Figures and Tables

**Algorithm 1:**
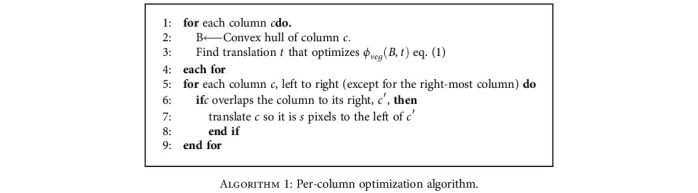
Per-column optimization algorithm.

**Algorithm 2:**
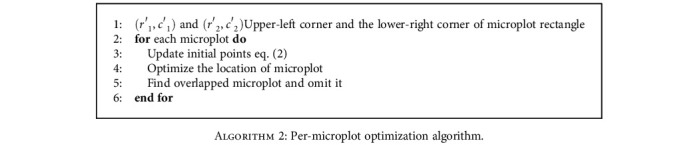
Per-microplot optimization algorithm.

**Algorithm 3:**
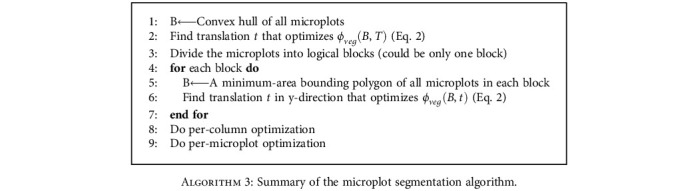
Summary of the microplot segmentation algorithm.

**Table 1a:** (a) The results of DSC and median of displacement error after using per-block, per-column, and per-microplot optimization steps for the canola dataset

Images	Mean microplot DSC			Median of displacement error		
	After per-block optimization step	After per-column optimization step	After per-microplot optimization step	After per-block optimization step	After per-column optimization step	After per-microplot optimization step
2017-06-28	87.97	91.61	90.89	1.13	0.62	0.65
2017-07-03	84.30	86.43	93.04	1.18	0.56	0.29
2017-07-07	78.43	78.70	88.46	1.71	1.64	0.51
2017-07-19	85.40	90.22	95.11	0.76	0.56	0.40
2017-07-26	92.32	90.59	94.72	0.39	0.46	0.40
2017-08-01	89.15	91.85	92.06	0.79	0.41	0.46
2017-08-09	89.91	90.07	92.58	0.56	0.57	0.47
2017-08-12	90.18	88.30	89.93	0.67	0.74	0.66
2017-08-16	81.62	85.68	82.84	1.03	0.91	1.09
2017-08-22	86.47	89.90	91.43	1.33	0.68	0.60
Mean	86.58	88.34	91.15	0.86	0.72	0.51

**Table 1b:** (b) The results of DSC and correctly identified microplots for the canola dataset

Images	Mean microplot DSC	Number of undetected microplots (DSC < 0.5)	Percentage of detected microplots (DSC ≥ 0.5)
	Single block	Single block	Single block
2017-06-28	90.89	0	100%
2017-07-03	93.04	1	99.57%
2017-07-07	88.46	1	99.57%
2017-07-19	95.11	0	100%
2017-07-26	94.72	0	100%
2017-08-01	92.06	0	100%
2017-08-09	92.58	0	100%
2017-08-12	89.93	0	100%
2017-08-16	82.84	4	98.29%
2017-08-22	91.43	0	100%
Mean	91.15	0.6	99.74%

**Table 2a:** (a) The results of DSC and median of displacement error after using per-block, per-column, and per-microplot optimization steps for the wheat dataset using a single block and three blocks in the per-block optimization step

Images	Mean microplot DSC			Median of displacement error		
	After per-block optimization step	After per-column optimization step	After per-microplot optimization step	After per-block optimization step	After per-column optimization step	After per-microplot optimization step
2018-03-07	62.62	72.81	91.79	1.79	0.79	0.34
2018-03-14	60.66	78.62	92.46	1.68	0.72	0.33
2018-04-04	58.59	60.99	90.39	2.10	1.50	0.34
2018-04-17	58.52	63.11	90.33	1.85	1.38	0.37
2018-04-25	59.77	70.38	92.06	2.17	1.10	0.35
2018-05-02	55.87	62.72	85.68	8.97	7.87	0.56
2018-05-08	57.26	65.07	84.46	7.74	5.68	0.62
Mean	59.04	67.67	89.56	3.76	2.72	0.42

**Table 2b:** (b) The results of DSC and correctly identified microplots for the wheat dataset using a single block and three blocks in the per-block optimization step

Images	Mean microplot DSC		Median of displacement error		Number of undetected microplots (DSC < 0.5)		Percentage of detected microplots (DSC ≥ 0.5)	
	Single block	Three blocks	Single block	Three blocks	Single block	Three blocks	Single block	Three blocks
2018-03-07	89.29	91.79	0.37	0.34	39	0	93.09%	100%
2018-03-14	92.36	92.46	0.33	0.33	0	0	100%	100%
2018-04-04	86.12	90.39	0.87	0.34	270	11	52.13%	98.05%
2018-04-17	89.50	90.33	0.4	0.37	8	3	98.58%	99.47%
2018-04-25	91.37	92.06	0.36	0.35	7	0	98.76%	100%
2018-05-02	82.17	85.68	0.6	0.56	56	7	90.08%	98.76%
2018-05-08	80.33	84.46	0.7	0.64	72	19	87.23%	96.63%
Mean	87.30	89.56	0.52	0.42	64.57	5.7	88.55%	98.99%

## Data Availability

The data is available upon request.

## References

[1] Araus J. L., Cairns J. E. (2014). Field high-throughput phenotyping: the new crop breeding frontier. *Trends in Plant Science*.

[2] Chawade A., van Ham J., Blomquist H., Bagge O., Alexandersson E., Ortiz R. (2019). High-throughput field-phenotyping tools for plant breeding and precision agriculture. *Agronomy*.

[3] Xu R., Li C., Paterson A. H. (2019). Multispectral imaging and unmanned aerial systems for cotton plant phenotyping. *PLoS One*.

[4] Guo W., Zheng B., Potgieter A. B. (2018). Aerial imagery analysis|quantifying appearance and number of sorghum heads for applications in breeding and agronomy. *Frontiers in plant science*.

[5] Duan T., Zheng B., Guo W., Ninomiya S., Guo Y., Chapman S. C. (2017). Comparison of ground cover estimates from experiment plots in cotton, sorghum and sugarcane based on images and ortho-mosaics captured by UAV. *Functional Plant Biology*.

[6] Barry P., Coakley R. (2013). FIELD Accuracy TEST of RPAS photogrammetry. *International Archives of the Photogrammetry, Remote Sensing and Spatial Information Sciences*.

[7] Haghighattalab A., Perez L. G., Mondal S. (2016). Application of unmanned aerial systems for high throughput phenotyping of large wheat breeding nurseries. *Plant Methods*.

[8] Hearst A. A., Cherkauer K. A. (2015). Research Article: Extraction of small spatial plots from geo-registered UAS imagery of crop fields. *Environmental Practice*.

[9] Recio J., Hermosilla T., Ruiz L., Palomar J. (2013). Automated extraction of tree and plot-based parameters in citrus orchards from aerial images. *Computers and Electronics in Agriculture*.

[10] Khan Z., Miklavcic S. J. (2019). An automatic field plot extraction method from aerial orthomosaic images. *Frontiers in plant science*.

[11] Tresch L., Mu Y., Itoh A. (2019). Easy MPE: extraction of quality microplot images for UAV-based high-throughput field phenotyping. *Plant Phenomics*.

[12] Matias F. I., Caraza-Harter M. V., Endelman J. B. (2020). FieldimageR: an R package to analyze orthomosaic images from agricultural field trials. *The Plant Phenome Journal*.

[13] Robb C., Hardy A., Doonan J. H., Brook J. (2020). Semi-automated field plot segmentation from UAS imagery for experimental agriculture. *Frontiers in Plant Science*.

[14] Ahmed I., Eramian M., Ovsyannikov I. Automatic detection and segmentation of lentil crop breeding plots from multi-spectral images captured by UAV-mounted camera.

[15] Chen C. J., Zhang Z. (2020). Grid: a python package for field plot phenotyping using aerial images. *Remote Sensing*.

[16] Meyer G. E., Neto J. C. (2008). Verification of color vegetation indices for automated crop imaging applications. *Computers and Electronics in Agriculture*.

[17] Woebbecke D. M., Meyer G. E., von Bargen K., Mortensen D. A. (1995). Color indices for weed identification under various soil, residue, and lighting conditions. *Transactions of the ASAE*.

[18] Storn R., Price K. (1997). Differential evolution | a simple and efficient heuristic for global optimization over continuous spaces. *Journal of Global Optimization*.

[19] Dice L. R. (1945). Measures of the amount of ecologic association between species. *Ecology*.

[20] Mardanisamani S., Maleki F., Hosseinzadeh Kassani S. Crop lodging prediction from UAV-acquired images of wheat and canola using a DCNN augmented with handcrafted texture features.

[21] Lottes P., Behley J., Chebrolu N., Milioto A., Stachniss C. (2020). Robust joint stem detection and crop-weed classification using image sequences for plant-specific treatment in precision farming. *Journal of Field Robotics*.

[22] Reza M. N., Na I. S., Baek S. W., Lee K.-H. (2019). Rice yield estimation based on K-means clustering with graph-cut segmentation using low-altitude UAV images. *Biosystems Engineering*.

